# 4-[(1*E*)-({[(Benzyl­sulfan­yl)methane­thio­yl]amino}­imino)­meth­yl]benzene-1,3-diol chloro­form hemisolvate: crystal structure, Hirshfeld surface analysis and computational study

**DOI:** 10.1107/S2056989020007070

**Published:** 2020-06-02

**Authors:** Nadia Liyana Khairuanuar, Karen A. Crouse, Huey Chong Kwong, Sang Loon Tan, Edward R. T. Tiekink

**Affiliations:** a Department of Chemistry, Faculty of Science, Universiti Putra Malaysia, UPM, Serdang 43400, Malaysia; b Research Centre for Crystalline Materials, School of Science and Technology, Sunway University, 47500 Bandar Sunway, Selangor Darul Ehsan, Malaysia

**Keywords:** crystal structure, Schiff base, hydrazine carbodi­thio­ate, hydrogen bonding, Hirshfeld surface analysis, DFT

## Abstract

The title hydrazine carbodi­thio­ate chloro­form hemisolvate, 2C_15_H_14_N_2_O_2_S_2_·CHCl_3_, comprises two independent hydrazine carbodi­thio­ate mol­ecules, *A* and *B*, and a chloro­form mol­ecule; the latter is statistically disordered about its mol­ecular threefold axis. The common features of the organic mol­ecules include an almost planar, central CN_2_S_2_ chromophore [r.m.s. deviation = 0.0203 Å (*A*) and 0.0080 Å (*B*)], an *E* configuration about the imine bond and an intra­molecular hydroxyl-O—H⋯N(imine) hydrogen bond. The major conformational difference between the mol­ecules is seen in the relative dispositions of the phenyl rings as indicated by the values of the dihedral angles between the central plane and phenyl ring of 71.21 (6)° (*A*) and 54.73 (7)° (*B*). Finally, a difference is seen in the disposition of the outer hydroxyl-H atoms, having opposite relative orientations. In the calculated gas-phase structure, the entire mol­ecule is planar with the exception of the perpendicular phenyl ring. In the mol­ecular packing, the *A* and *B* mol­ecules assemble into a two-mol­ecule aggregate *via* N—H⋯S hydrogen bonds and eight-membered {⋯HNCS}_2_ synthons. The dimeric assemblies are connected into supra­molecular chains *via* hydroxyl-O—H⋯O(hydrox­yl) hydrogen bonds and these are linked into a double-chain through hy­droxy-O—H⋯π(phen­yl) inter­actions. The double-chains are connected into a three-dimensional architecture through phenyl-C—H⋯O(hydrox­yl) and phenyl-C—H⋯π(phen­yl) inter­actions. The overall assembly defines columns along the *a*-axis direction in which reside the chloro­form mol­ecules, which are stabilized by chloro­form–methine-C—H⋯S(thione) and phenyl-C—H⋯Cl contacts. The analysis of the calculated Hirshfeld surfaces, non-covalent inter­action plots and inter­action energies confirm the importance of the above-mentioned inter­actions, but also of cooperative, non-standard inter­actions such as π(benzene)⋯π(hydrogen-bond-mediated-ring) contacts.

## Abstract 2

The title hydrazine carbodi­thio­ate chloro­form hemi-solvate features approximately planar mol­ecules with splayed phenyl groups. Supra­molecular tapes are formed in the crystal through a combination of N—H⋯S, hydroxyl-O—H⋯O(hydrox­yl) and hydroxyl-O—H⋯π(phen­yl) inter­actions.

## Chemical context   

Schiff bases are ketone or aldehyde analogues in which the carbonyl group (C=O) is replaced by an azomethine group (C=N). Di­thio­carbazato Schiff bases have received considerable attention because of the presence of both soft sulfur and hard nitro­gen atoms (Mohamed *et al.*, 2009 ▸), which enables them to readily form complexes with transition metals in different oxidation states (Centore *et al.*, 2013 ▸). Di­thio­carbazato Schiff bases and their metal complexes show a wide range of anti-bacterial (da Silva *et al.*, 2011 ▸), anti-fungal (Nazimuddin *et al.*, 1992 ▸), anti-viral (Pandeya *et al.*, 1999 ▸) and anti-malarial (Dutta *et al.*, 2006 ▸) activities. In addition, some di­thio­carbazate derivatives display cytotoxicity towards a variety of cancer cell lines (Yusof *et al.*, 2020 ▸) and some exhibit varying degrees of analgesic and anti-inflammatory activities (Zangrando *et al.*, 2015 ▸).
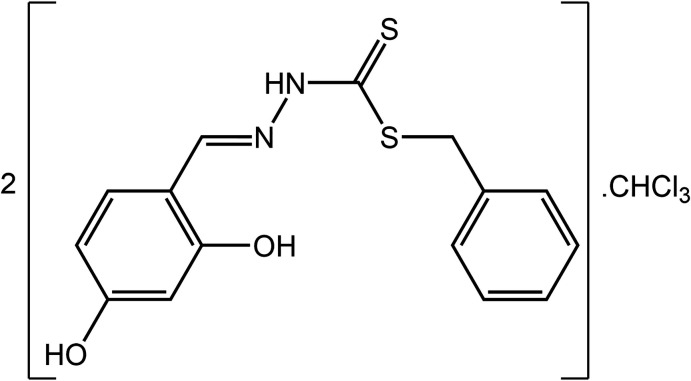


As part of on-going studies in this area (Rusli *et al.*, 2020 ▸), herein the synthesis and X-ray crystal structure determination of the title compound, C_15_H_14_N_2_O_2_S_2_·0.5CHCl_3_, (I), is described. The experimental study is complemented by an analysis of the calculated Hirshfeld surfaces along with some computational chemistry.

## Structural commentary   

**Figure 1:**
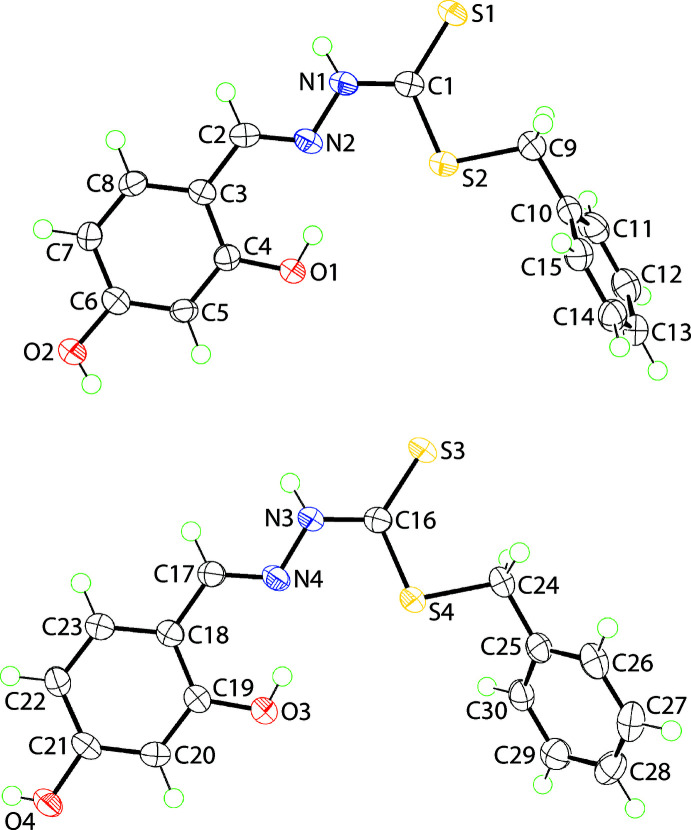
The mol­ecular structures of the two independent hydrazine carbodi­thio­ate mol­ecules in (I) showing the atom-labelling scheme and displacement ellipsoids at the 70% probability level.

The crystallographic asymmetric unit of (I) comprises two independent hydrazine carbodi­thio­ate mol­ecules and a chloro­form solvent mol­ecule of crystallization, with the latter disordered statistically about its mol­ecular threefold axis. The mol­ecular structures of the organic mol­ecules are shown in Fig. 1 ▸ and selected geometric parameters are collected in Table 1 ▸. The central CN_2_S_2_ atoms define an almost planar residue, exhibiting an r.m.s. deviation of 0.0203 Å with maximum deviations to either side of the plane of 0.0264 (12) Å, for the N2 atom, and 0.0319 (16) Å for N1; the C2 and C9 atoms lie, respectively, 0.161 (3) and 0.096 (4) Å out of the plane, in the direction of the N2 atom. The comparable plane for the S3-mol­ecule is significantly more planar with an r.m.s. deviation = 0.0080 Å with maximum deviations of 0.0131 (16) Å for the N3 atom and 0.0104 (12) Å for atom N4; the C17 atom lies 0.018 (3) Å out of the central plane in the direction of the N3 atom, and the C24 lies 0.123 (3) Å out of the plane in the direction of the N4 atom. The small difference in planarity is reflected in the C1—N1—N2—C2 and C16—N3—N4—C17 torsion angles of 171.8 (2) and 179.3 (2)°, respectively. More significant conformational differences are apparent in rest of the mol­ecules: for the S1-mol­ecule, the dihedral angles between the central residue and terminal hy­droxy­benzene and phenyl rings are 6.18 (13) and 77.21 (6)°, respectively, indicating close to co-planar and perpendicular relationships; the dihedral angle between the terminal rings is 71.22 (8)°. The equivalent dihedral angles for the S3-mol­ecule are 6.07 (13), 54.53 (6) and 54.73 (7)°, respectively. The other notable difference between the mol­ecules relates to the relative orientation of the hy­droxy-H atoms in the 4-position, no doubt arising owing to the dictates of the mol­ecular packing.

The relatively co-planar relationship between the central residue and the appended hy­droxy­benzene ring allows for the formation of an intra­molecular hy­droxy-O—H⋯N(imine) hydrogen bond in each mol­ecule, Table 2 ▸. The configuration about the imine bond is *E* in each case. The comparison of geometric parameters in Table 1 ▸ shows a high degree of concordance. The C=S bonds are significantly shorter than the other C—S bonds and this impacts upon the angles subtended at the C1 atom, being wider for those involving the thione-S atoms, and with the widest angle involving the two sulfur atoms.

## Theoretical mol­ecular structure   

The two independent mol­ecules of the hydrazine carbodi­thio­ate ester in (I) were subjected to gas-phase geometry optimization calculations using the density functional wB97XD level of theory (Chai & Head-Gordon, 2008 ▸) and the Def2TZVP basis set (Weigend & Ahlrichs, 2005 ▸) as available in *Gaussian16* (Frisch *et al.*, 2016 ▸). Selected geometric data for the optimized structure are included in Table 1 ▸ for comparison with the experimental mol­ecular structures.

**Figure 2:**
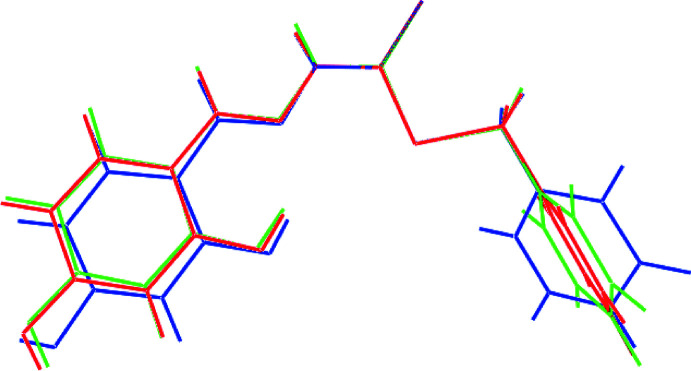
An overlay diagram of the two independent hydrazine carbodi­thio­ate mol­ecules in (I): S1-mol­ecule (red image) and S3-mol­ecule (blue), and geometry optimized structure (green). The mol­ecules have been overlapped so the CS_2_ residues are coincident.

An overlay diagram for the experimental and theoretical, gas-phase structures is shown in Fig. 2 ▸. From here, the conformational differences between the two experimental structures are highlighted, especially the relative disposition of the terminal hy­droxy­benzene and phenyl rings. The geometric parameters extracted from the gas-phase structure reflect expectation but there are considerable conformational differences. Free from the restrictions of the crystalline manifold, the optimized structure is planar with the exception of the phenyl ring, which lies in a position perpendicular to the rest of the mol­ecule. It is inter­esting to note that, qualitatively, the overall conformation in the S1-mol­ecule more closely matches the gas-phase structure compared to the S3-mol­ecule. This is reflected in the relative adjustments in the torsion angles, such as in the S2—C9—C10—C11, C15 torsion angles, Table 1 ▸.

## Supra­molecular features   

**Figure 3:**
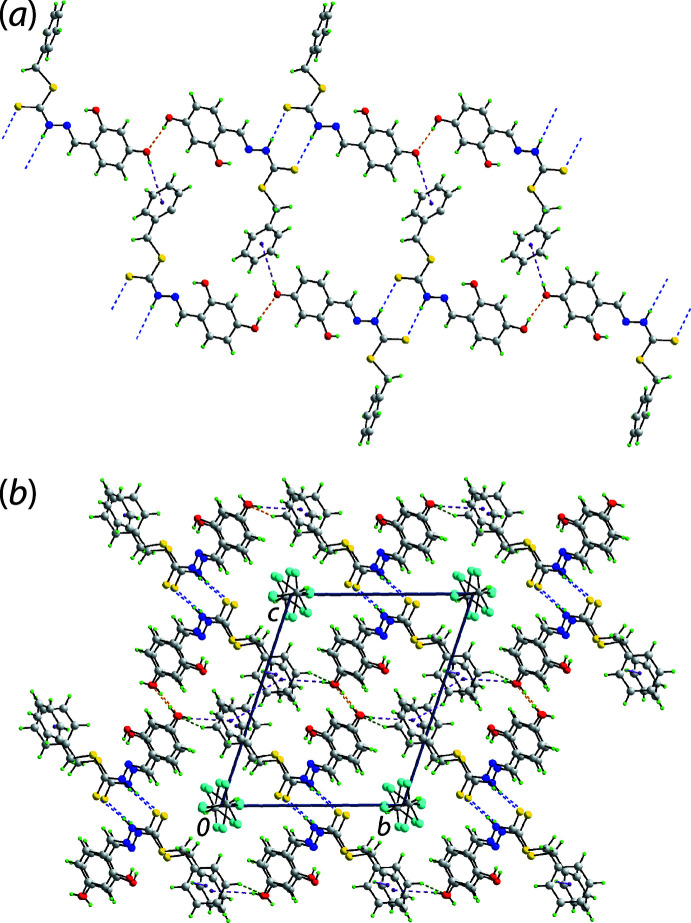
Mol­ecular packing in (I): (*a*) the linear, supra­molecular double-chain in which dimeric aggregates sustained by thio­amide-N—H⋯S(thio­amide) hydrogen bonding, shown as blue dashed lines, are connected by hydroxyl-O—H⋯O(hydrox­yl) (orange) and hydroxyl-O—H⋯π(phenyl) inter­actions (purple) and (*b*) a view of the unit-cell contents shown in projection down the *a* axis highlighting the three-dimensional framework and columns, parallel to the *a*-axis, in which reside the disordered CHCl_3_ mol­ecules. The phenyl-C—H⋯O(hydrox­yl) and phenyl-C—H⋯π(phen­yl) inter­actions are shown as green and pink dashed lines, respectively.

In the mol­ecular packing, the independent hydrazine carbodi­thio­ate mol­ecules are connected by thio­amide-N—H⋯S(thione) hydrogen bonds to form a two-mol­ecule aggregate. The O2-hydroxyl H atom forms a hydrogen bond with the hydroxyl-O4 atom, connecting the dimeric aggregates into a supra­molecular chain. Centrosymmetrically related chains are connected into a double-chain *via* O4-hy­droxy-O—H⋯π(phen­yl) inter­actions as illustrated in Fig. 3 ▸(*a*). The assembly lies parallel to [2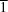 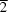]. The connections between the double-chains that form a three-dimensional architecture are of the type phenyl-C—H⋯O(hy­droxy) and phenyl-C—H⋯π(phen­yl). This architecture defines columns, parallel to the *a*-axis direction, which accommodate the chloro­form mol­ecules, Fig. 3 ▸(*b*). The links between the host scaffold and the chloro­form mol­ecules are of the type methine-C—H⋯S(thione) and phenyl-C—H⋯Cl, as detailed in Table 2 ▸.

## Analysis of the Hirshfeld surfaces   

**Figure 4:**
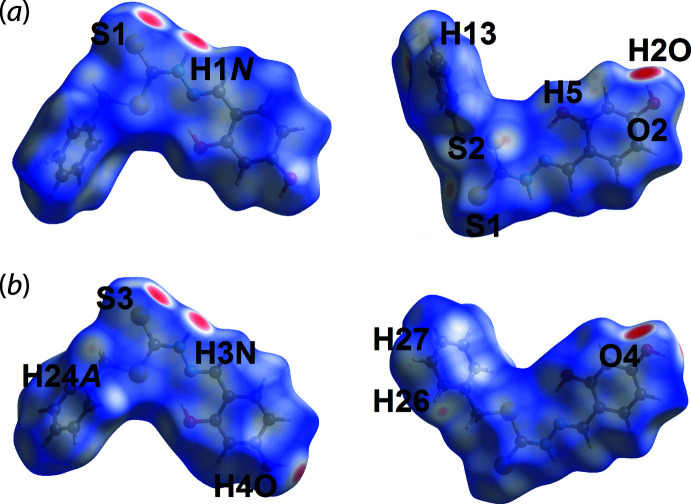
Views of the Hirshfeld surface for (I) mapped over *d*_norm_ for the (*a*) S1-containing mol­ecule and (*b*) S3-mol­ecule. The surfaces were mapped in the range −0.572 to +1.067 arbitrary units.

**Figure 5:**
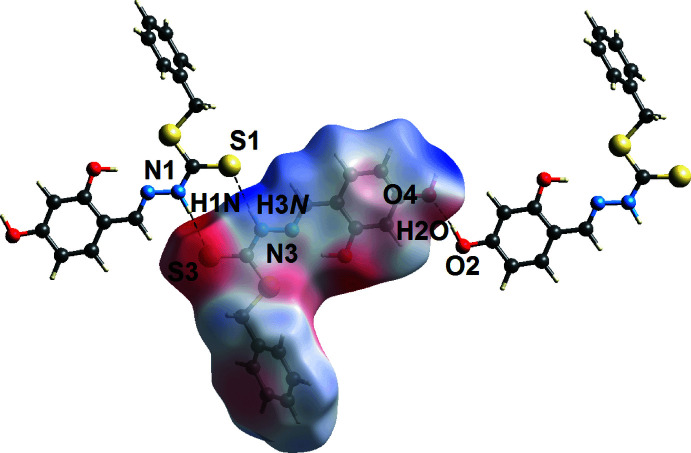
A view of the Hirshfeld surface mapped over the electrostatic potential for the S3-containing mol­ecule in the range −0.055 to +0.134 a.u. The red and blue regions represent negative and positive electrostatic potentials, respectively.

**Figure 6:**
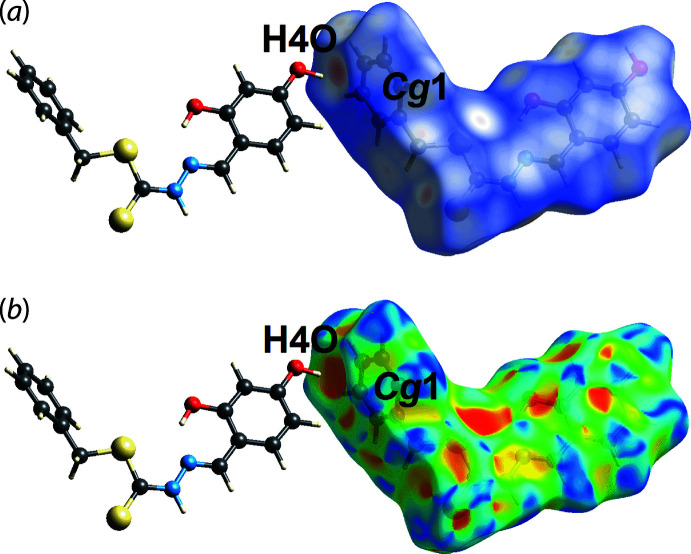
A view of the Hirshfeld surface mapped over (*a*) *d*_norm_ and (*b*) the shape-index property highlighting the inter­molecular hydroxyl-O—H⋯π(phen­yl) contacts as red and dark-orange regions, respectively.

The calculation of the Hirshfeld surfaces for (I) were conducted following literature procedures (Tan *et al.*, 2019 ▸) employing *CrystalExplorer17* (Turner *et al.*, 2017 ▸) in order to reveal further details of the supra­molecular association in the crystal. Calculations were performed on overall (I) and the individual S1- and S3-di­thio­carbazate mol­ecules. That the thio­amide and hy­droxy­benzene residues play a crucial role in the formation of directional inter­actions is indicated by the dark-red spots observed near the participating atoms on the Hirshfeld surfaces of the S1- and S3-containing mol­ecules in Fig. 4 ▸. These observations are further confirmed by electrostatic potential mapping in which the N—H⋯S and O—H⋯O hydrogen bonds are shown as dark-blue (electropositive) and dark-red (electronegative) regions in Fig. 5 ▸. In the *d*_norm_-surface mapping, some additional inter­actions corresponding to contacts listed in Table 3 ▸ are indicated by light-red spots around both di­thio­carbazate mol­ecules in Fig. 4 ▸. No significant contacts are indicated on the *d*_norm_-mapped surfaces for the disorder components of the chloro­form mol­ecule (not shown). The O4—H4*O*⋯π(C10–C15) inter­action is visible through *d*_norm_ surface mapping in Fig. 6 ▸(*a*) and shape-index surface mapping in Fig. 6 ▸(*b*).

**Figure 7:**
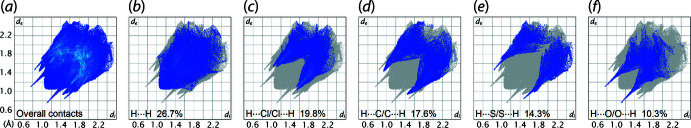
(*a*) The full two-dimensional fingerprint plot for (I) and fingerprint plots delineated into (*b*) H⋯H, (*c*) H⋯Cl/Cl⋯H, (*d*) H⋯C/C⋯H, (*e*) H⋯S/S⋯H and (*f*) H⋯O/O⋯H contacts.

As illustrated in Fig. 7 ▸(*a*), the overall two-dimensional fingerprint plot of (I) shows characteristic pseudo-symmetric wings along the *d*_e_ and *d*_i_ diagonal axes. This plot has also been delineated into H⋯H, H⋯Cl/Cl⋯H, H⋯C/C⋯H, H⋯S/S⋯H and H⋯O/O⋯H contacts as illustrated in Fig. 7 ▸(*b*)–(*f*); the percentage contributions to the Hirshfeld surface from different inter­atomic contacts are summarized in Table 4 ▸ for overall (I) and the individual S1- and S3-mol­ecules.

The greatest contribution to the overall surface is from H⋯H contacts with the shortest contact, manifested in the peak tipped at *d*_e_ + *d*_i_ ∼2.2 Å corresponding to the H5⋯H13 contact listed in Table 3 ▸. The next most prominent contacts are due to H⋯Cl/Cl⋯H surface contacts reflecting generally weak contacts involving the solvent chloro­form mol­ecule, Tables 2 ▸ and 3 ▸. The H⋯C/C⋯H contacts on the Hirshfeld surface (17.6% of the overall contribution) partly reflect the O—H⋯π contacts as discussed above. The significant contributions from H⋯S/S⋯H (14.3%) and H⋯O/O⋯H (10.3%) contacts reflect the presence of the N—H⋯S and O—H⋯O hydrogen bonds. These appear as two sharp symmetric spikes in the fingerprint plots at *d*_e_ + *d*_i_ ∼2.3 and 1.9 Å, respectively in Fig. 7 ▸(*e*) and (*f*). For overall (I), the sum of the percentage contributions from the other 16 different contacts, all of which occur at separations greater than the sum of the respective van der Waals radii, is less than 14%.

Hirshfeld surface analysis can also be extremely useful for distinguishing between/confirming the presence of multiple mol­ecules in the asymmetric unit (Jotani *et al.*, 2019 ▸). The percentage contributions to the Hirshfeld surfaces for the S1- and S3-mol­ecules in (I) are included in Table 3 ▸. The major difference in the percentage contributions between overall (I) and the individual S1- and S3-mol­ecules rests with the H⋯Cl/Cl⋯H inter­actions. These are approximately half for the latter, reflecting the fact that the chloro­form mol­ecule forms close to equal contributions to the surface contacts of the individual S1- and S3-mol­ecules. The distinguishing features between the S1- and S3-mol­ecules relate to the increased percentage contribution of H⋯O/O⋯H contacts for the former, reflecting the C27—H27⋯O2 contact for which there is no equivalent for the S3-mol­ecule, and also the increased H⋯Cl/Cl⋯H contacts for the S3-mol­ecule, reflecting the H⋯Cl contacts this mol­ecule forms with the chloro­form mol­ecule.

## Computational chemistry   

**Figure 8:**
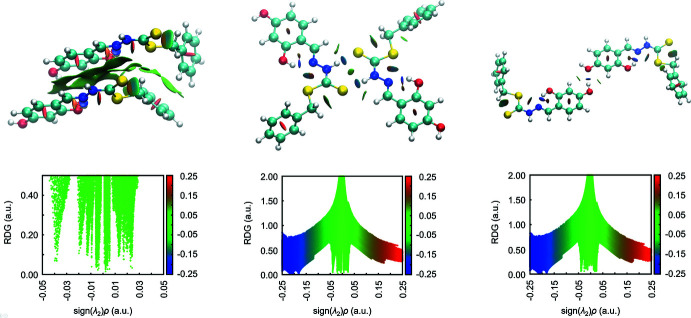
The non-covalent inter­action plot and corresponding RDG *versus* sign(λ_2_)ρ(*r*) plots for the dimeric aggregates sustained by (*a*) a combination of π(C3–C8)–quasi-π(N4,C17–C19,O3,H3*O*), quasi-π(N2,C2–C4,O1,H1*O*)–quasi-π(S4,C16,N3,N4), C24—H24*A*⋯S2, C14–H14⋯π(C25–C30) and C15—H15⋯\p(C25–C30) inter­actions between S1- and S3-mol­ecules, (*b*) N1—H1*N*⋯S3 and N3—H3*N*⋯S1 hydrogen bonds and (*c*) O2—H2*O*⋯O4 inter­actions.

Several of the non-covalent inter­actions present in (I) were qualitatively evaluated using *NCIPLOT* (Johnson *et al.*, 2010 ▸) by verifying the strength of an inter­action through visualization of the gradient isosurface based on the electron density derivatives obtained from wavefunction calculations (Contreras-García *et al.*, 2011 ▸). Apart from the described contacts detected through Hirshfeld surface analyses, some additional non-covalent inter­actions were verified using NCI plots. These include the relatively large localized green domain observed between the hy­droxy­benzene fragment of the S1-mol­ecule that extends towards the azomethine group of the S3-mol­ecule, Fig. 8 ▸(*a*), indicating a weak inter­action; overall sign(λ_2_)ρ < −0.05 a.u. This may arise from a π–π inter­action between the hy­droxy­benzene ring of the S1-mol­ecule and the quasi-(N4,C17–C19,O3,H3*O*) aromatic ring of the S3-mol­ecule. The ability of quasi-π-systems, where the ring is closed by a hydrogen bond, to engage in such inter­actions (Calvin & Wilson, 1945 ▸; Karabıyık *et al.*, 2014 ▸), including when one of the constituent atoms is a metal atom (Yeo *et al.*, 2014 ▸), has been established in the literature. There is also evidence of weakly attractive regions correlating with inter­actions between the π-systems of the (N2,C2–C4,O1,H1*O*) and (S4,C16,N3,N4) residues along with C24—H24*A*⋯S2 and C14—H14, C15—H15⋯π(C25–C30) contacts.

Among all close contacts present in (I), the pairwise N1—H1*N*⋯S3/N3—H3*N*⋯S1 and O2—H2*O*⋯O4 inter­actions exhibit a blue, *i.e.* strongly attractive, isosurface between the corresponding points of contact having a density values [sign(λ_2_)ρ] more than −0.18 a.u., Fig. 6 ▸(*b*) and (*c*). The intra­molecular O—H⋯N contacts reveal similar attractive inter­actions.

To complement the *NCIPLOT* results, the strength of inter­action for each close contact was qu­anti­fied by calculation of the inter­action energy in *Gaussian16* (Frisch *et al.*, 2016 ▸). All pairwise inter­actions were submitted for gas-phase energy calculation by the long-range corrected ωB97XD functional combining the D2 version of Grimme’s dispersion model (Chai & Head-Gordon, 2008 ▸) with Ahlrichs’ valence triple-zeta polarization basis sets (ωB97XD/def2-TZVP) (Weigend & Ahlrichs, 2005 ▸), for which the dispersion model has been demonstrated to give better accuracy in inter­action energy as compared to other computationally expensive models (Andersen *et al.*, 2014 ▸). Counterpoise methods (Boys & Bernardi, 1970 ▸; Simon *et al.*, 1996 ▸) were applied to correct for basis set superposition error (BSSE) in all calculated energies.

Referring to Fig. 8 ▸(*a*), the combination of π(C3–C8)–quasi-π(N4,C17–C19,O3,H3*O*), quasi-π(N2,C2–C4,O1,H1*O*)–quasi-π(S4,C16,N3,N4), C24—H24*A*⋯S2, C14—H14⋯ π(C25–C30) and C15—H15⋯π(C25–C30) between S1- and S3-mol­ecules exhibits the greatest inter­action energy among all close contacts with an *E* of −65.73 kJ mol^−1^, Table 5 ▸. This energy slightly exceeds that exhibited by the eight-membered {⋯HNCS}_2_ synthon, being the second strongest inter­action with *E* = −59.79 kJ mol^−1^. The strength of the N1—H1*N*⋯S3/N3–H3*N*⋯S1 inter­action is consistent with the energy range of −54.06 to −57.99 kJ mol^−1^ displayed by the equivalent contacts in the cinnamaldehyde Schiff base of *S*-(4-methyl­benz­yl) di­thio­carbaza­tes calculated through wB97XD/6-31G(*d*,*p*) (Yusof *et al.*, 2017 ▸). Next, in terms of energy, is the C29—H29⋯ π(C10–C15) inter­action with *E* = −26.28 kJ mol^−1^, which is surprisingly higher than that of the more typical O2—H2*O*⋯O4 inter­action with an *E* value of −23.47 kJ mol^−1^. The energies of other inter­actions in the order of reducing strength are tabulated in Table 5 ▸.

## Database survey   

There are six literature precedents for X-ray crystal structure determinations of mol­ecules of the general formula (*n*-OH-benzene)C=NN(H)C(=S)S*R*, five of which have the hydroxyl substituent in the 2-position enabling the formation of an intra­molecular hy­droxy-O—H⋯N(imine) hydrogen bond. In the most closely related compounds, *i.e*. with 2-OH substituents, the *R* group in the ester substituent is methyl (CSD refcode LUDGIC; Madanhire *et al.*, 2015 ▸) and *n*-hexyl (TACYUU; Begum *et al.*, 2016 ▸). An inter­esting feature of the latter structure is the presence of four independent mol­ecules in the asymmetric unit. The other closely related structure has *R* = benzyl and also a meth­oxy group in the 3-position of the hy­droxy­benzene ring (EHIXUQ; Yusof *et al.*, 2016 ▸). Unlike the previous two mol­ecules, which are very close to being planar, the benzyl group is perpendicular to the plane through the rest of the mol­ecule. The three remaining structures have a methyl substituent at the imine-C atom. Two of these have 2-OH substituents in the benzene ring, one with *R* = benzyl (QUCLIL; Biswal *et al.*, 2015 ▸), with a twisted conformation, and the other with *R* = CH_2_=CH_2_ (NILRII; Lima *et al.*, 2018 ▸), being a planar mol­ecule. The sixth and final analogue is a 3-OH derivative with *R* = benzyl (LUBNIH; Zangrando *et al.*, 2015 ▸); this mol­ecule exhibits a twisted conformation in its crystal.

## Synthesis and crystallization   

Two solutions, *S*-benzyl­dithio­carbazate (5.0 g, 0.025 mol in 60 ml of hot ethanol) and 2,4-di­hydroxy­benzaldehyde (3.45 g, 0.025 mol in 25 ml ethanol) were mixed and heated until the initial volume was reduced by half. The yellow precipitate formed after cooling the mixture to room temperature was collected and washed with cold ethanol. It was recrystallized from ethanol solution and dried over silica gel for three days. Light-yellow prisms were obtained from its 1:1 diethyl ether/chloro­form solution by slow evaporation.

Yield: 4.98 g, 62%; m.p. 463–465 K. FT–IR UATR (solid), λ (cm^−1^): 3310 (O—H, *v*), 3094 (N—H, *v*), 1604 (C=N, *v*), 1100 (N—N, *v*), 1024 (C=S, *v*), 948 (S=C—S, *v*). ^1^H NMR (400 MHz, CDCl_3_): *δ* 13.19 (*s*, 1H, N—H), 10.21 (*s*, 1H, O—H), 10.07 (*s*, 1H, O—H), 8.35 (*s*, 1H, N=CH), 7.45 (*t*, 2H, *J* = 7.5 Hz, Ph), 7.36 (*t*, 2H, *J* = 7.5 Hz, Ph), 7.21 (*t*, 1H, *J* = 7.5 Hz, Ph), 6.29 (*s*, 1H, benzene), 6.26 (*d*, 2H, *J* = 4.0 Hz, benzene), 4.44 (*s*, 2H, CH_2_). ^13^C{^1^H}-NMR (100 MHz, CDCl_3_): *δ* ppm. 194.4 (C=S), 162.1, 159.6 (C—OH), 146.5 (N=C), 129.7–127.8 (Ph & benzene), 38.0 (CH_2_); GCMS (DI): *m*/*z* calculated for C_15_H_14_N_2_O_2_S_2_^+^ [*M* ^+^]: 318, found 318.

## Refinement   

Crystal data, data collection and structure refinement details are summarized in Table 6 ▸. The carbon-bound H atoms were placed in calculated positions (C—H = 0.95–1.00 Å) and were included in the refinement in the riding-model approximation, with *U*_iso_(H) set to 1.2*U*_eq_(C). The O- and N-bound H atoms were located in a difference-Fourier map but, were refined with O—H (0.84±0.01 Å) and N—H (0.88±0.01 Å) distance restraints, and with *U*_iso_(H) set to 1.5*U*_eq_(O) and to 1.2*U*_eq_(N), respectively. The CHCl_3_ solvent mol­ecule is statistically disordered about the mol­ecular threefold axis. The C31 atom is common to both conformations and the individual Cl atoms were refined anisotropically. A loose distance restraint for C—Cl was applied, *i.e*. C—Cl = 1.76±0.02 Å. The maximum and minimum residual electron density peaks of 1.04 and 1.22 e Å^−3^, respectively, are located 1.03 and 0.90 Å from the Cl3′ atom.

## Supplementary Material

Crystal structure: contains datablock(s) . DOI: 10.1107/S2056989020007070/hb7919sup1.cif

Structure factors: contains datablock(s) I. DOI: 10.1107/S2056989020007070/hb7919Isup2.hkl

Supporting information file. DOI: 10.1107/S2056989020007070/hb7919Isup3.cml

CCDC reference: 2005815

Additional supporting information:  crystallographic information; 3D view; checkCIF report

## Figures and Tables

**Table 1:** Selected geometric parameters (Å, °) in (I)

Parameter	S1-mol­ecule	S3-mol­ecule	Geometry-optimized
C1—S1	1.680 (3)	1.675 (2)	1.650
C1—S2	1.755 (3)	1.749 (3)	1.749
C9—S2	1.816 (3)	1.823 (3)	1.815
C1—N1	1.327 (3)	1.340 (3)	1.351
N1—N2	1.377 (3)	1.376 (3)	1.355
C2—N2	1.289 (3)	1.291 (3)	1.279
			
S1—C1—S2	124.88 (16)	124.25 (16)	126.6
S1—C1—N1	120.7 (2)	121.44 (19)	120.2
S2—C1—N12	114.43 (19)	114.31 (18)	113.2
C1—S2—C9	102.06 (13)	101.78 (12)	101.9
C1—N1—N2	120.7 (2)	119.5 (2)	123.0
N1—N2—C2	116.2 (2)	116.9 (2)	117.9
N2—C2—C3	121.5 (2)	121.1 (2)	122.7
			
S2–C9—C10—C11	97.7 (3)	−123.6 (2)	90.0
S2—C9—C10—C15	−81.2 (3)	57.9 (3)	−89.3
S1—C1—S2—C9	2.2 (2)	−3.8 (2)	0.0
S1—C1—N1—N2	−176.2 (2)	178.6 (2)	−179.9
S2—C1—N1—N2	3.9 (3)	−1.7 (3)	0.2
C1—N1—N2—C2	171.8 (2)	179.3 (2)	179.9
N1—N2—C2—C3	−178.8 (2)	179.2 (2)	−180.0
N2—C2—C3—C4	0.9 (4)	−3.4 (4)	0.0
N2—C2—C3—C8	−179.3 (2)	177.0 (2)	180.0

**Table 2:** Hydrogen-bond geometry (Å, °) *Cg*1 and *Cg*2 are the centroids of the (C10–C15) and (C25–C30) rings, respectively.

*D*—H⋯*A*	*D*—H	H⋯*A*	*D*⋯*A*	*D*—H⋯*A*
O1—H1*O*⋯N2	0.83 (3)	1.91 (3)	2.653 (3)	148 (3)
O3—H3*O*⋯N4	0.78 (4)	1.97 (4)	2.663 (3)	148 (4)
N1—H1*N*⋯S3^i^	0.88 (2)	2.46 (2)	3.323 (2)	168 (2)
N3—H3*N*⋯S1^i^	0.88 (2)	2.53 (2)	3.394 (2)	171 (2)
O2—H2*O*⋯O4^ii^	0.76 (4)	2.09 (4)	2.841 (3)	170 (4)
O4—H4*O*⋯*Cg*1^iii^	0.75 (4)	3.00 (4)	3.735 (3)	170 (4)
C27—H27⋯O2^iv^	0.95	2.59	3.206 (4)	122
C11—H11⋯*Cg*2^v^	0.95	2.91	3.541 (3)	125
C29—H29⋯*Cg*1^vi^	0.95	2.87	3.506 (3)	125
C26—H26⋯Cl1^vii^	0.95	2.75	3.488 (4)	135
C31—H31⋯Cl2^viii^	1.00	2.66	3.512 (4)	143
C31′—H31′⋯S1^i^	1.00	2.77	3.579 (4)	139

Symmetry codes: (i) 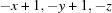; (ii) 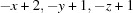; (iii) 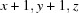; (iv) 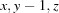; (v) 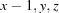; (vi) 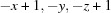; (vii) 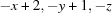; (viii) 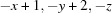.

**Table 3:** A summary of short inter­atomic contacts (Å) for (I)*^*a*^*

Contact	Distance	Symmetry operation
S1⋯H3*N^*b*^*	2.40	1 + *x*, 1 − *y*, −*z*
S3⋯H1*N^*b*^*	2.33	1 + *x*, 1 − *y*, −*z*
O4⋯H2*O^*b*^*	1.87	*x*, *y*, *z*
S1⋯H31	2.87	1 + *x*, 1 − *y*, −*z*
S1⋯H31′	2.71	1 + *x*, 1 − *y*, −*z*
S2⋯H24*A*	2.82	*x*, *y*, *z*
O2⋯H27	2.53	*x*, 1 + *y*, *z*
C22⋯H27	2.73	*x*, 1 + *y*, *z*
H5⋯H13	2.17	1 − *x*, −*y*, 1 − *z*
Cl1⋯H26	2.66	2 − *x*, 1 − *y*, −*z*

Notes: (*a*) The inter­atomic distances are calculated in *Crystal Explorer 17* (Turner *et al.*, 2017) with the *X*—H bond lengths are adjusted to their neutron values; (*b*) these inter­actions correspond to conventional hydrogen bonds.

**Table 4:** The percentage contributions of inter­atomic contacts to the Hirshfeld surface for (I) and for the S1- and S3-mol­ecules

Contact	Percentage contribution		
	(I)	S1-mol­ecule	S3-mol­ecule
H⋯H	26.7	29.7	27.6
H⋯Cl/Cl⋯H	19.8	8.0	11.3
H⋯C/C⋯H	17.6	21.8	23.0
H⋯S/S⋯H	14.3	14.8	14.2
H⋯O/O⋯H	10.3	12.1	10.0
Others	11.3	13.6	13.9

**Table 5:** A summary of inter­action energies (kJ mol^−1^) calculated for (I)

Contact	Inter­action Energy, *E* ^BSSE^_int_	symmetry operation
π(C3–C8)⋯quasi-π(N4,C17–C19,O3,H3*O*) +		
quasi-π(N2,C2–C4,O1,H1*O*)⋯quasi-π(S4,C16,N3,N4)′		
C24—H24*A*⋯S2 +		
C14—H14⋯π(C25–C30) +		
C15—H15⋯π(C25–C30)	−65.73	*x*, *y*, *z*
N1—H1*N*⋯S3 +		
N3—H3*N*⋯S1	−59.79	1 − *x*, 1 − *y*, − *z*
C29—H29⋯π(C10–C15)	−26.28	1 − *x*, − *y*, 1 − *z*
O2—H2*O*⋯O4	−23.47	2 − *x*, 1 − *y*, 1 − *z*
C5—H5⋯π(C10–C15) +		
C13—H13⋯π(C3–C8)	−20.08	1 − *x*, − *y*, 1 − *z*
O4—H4*O*⋯π(C10–C15)	−19.72	1 + *x*, 1 + *y*, *z*
C31′—H31′⋯S1	−14.39	1 − *x*, 1 − *y*, − *z*
C31—H31⋯S1	−13.22	1 − *x*, 1 − *y*, − *z*
C31—H31⋯Cl2	−10.25	1 − *x*, 2 − *y*, − *z*
C27—H27⋯π(C18–C23)	−9.84	*x*, −1 + *y*, *z*
C26—H26⋯Cl1	−5.27	2 − *x*, 1 − *y*, − *z*
C27—H27⋯O2	−4.68	*x*, −1 + *y*, *z*

**Table 6:** Experimental details

Crystal data	
Chemical formula	2C_15_H_14_N_2_O_2_S_2_·CHCl_3_
*M*_r_	756.17
Crystal system, space group	Triclinic, *P* 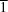
Temperature (K)	100
*a*, *b*, *c* (Å)	9.3193 (5), 12.7525 (7), 15.7294 (8)
α, β, γ (°)	68.712 (5), 74.217 (5), 76.098 (5)
*V* (Å^3^)	1655.13 (17)
*Z*	2
Radiation type	Cu *K*α
μ (mm^−1^)	5.23
Crystal size (mm)	0.11 × 0.09 × 0.03

Data collection	
Diffractometer	Oxford Diffraction Xcalibur, Eos, Gemini
Absorption correction	Multi-scan (*CrysAlis RED*; Oxford Diffraction, 2006 ▸)
*T*_min_, *T*_max_	0.766, 1.000
No. of measured, independent and observed [*I* > 2σ(*I*)] reflections	24002, 6572, 5466
*R*_int_	0.034
(sin θ/λ)_max_ (Å^−1^)	0.622

Refinement	
*R*[*F* ^2^ > 2σ(*F* ^2^)], *wR*(*F* ^2^), *S*	0.051, 0.136, 1.03
No. of reflections	6572
No. of parameters	461
No. of restraints	9
H-atom treatment	H atoms treated by a mixture of independent and constrained refinement
Δρ_max_, Δρ_min_ (e Å^−3^)	1.04, −1.22

Computer programs: *CrysAlis CCD* and *CrysAlis RED* (Oxford Diffraction, 2006 ▸), *SHELXT2014/4* (Sheldrick, 2015*a* ▸), *SHELXL2018/3* (Sheldrick, 2015*b* ▸), *ORTEP-3 for Windows* (Farrugia, 2012 ▸), *DIAMOND* (Brandenburg, 2006 ▸) and *publCIF* (Westrip, 2010 ▸).

## supplementary crystallographic information

### Crystal data

**Table:**

2C_15_H_14_N_2_O_2_S_2_·CHCl_3_	*Z* = 2
*M**_r_* = 756.17	*F*(000) = 780
Triclinic, *P*1	*D*_x_ = 1.517 Mg m^−^^3^
*a* = 9.3193 (5) Å	Cu *K*α radiation, λ = 1.54178 Å
*b* = 12.7525 (7) Å	Cell parameters from 9024 reflections
*c* = 15.7294 (8) Å	θ = 3.1–73.3°
α = 68.712 (5)°	µ = 5.23 mm^−^^1^
β = 74.217 (5)°	*T* = 100 K
γ = 76.098 (5)°	Prism, yellow
*V* = 1655.13 (17) Å^3^	0.11 × 0.09 × 0.03 mm

### Data collection

**Table:**

Oxford Diffraction Xcalibur, Eos, Gemini diffractometer	6572 independent reflections
Radiation source: Enhance (Cu) X-ray Source	5466 reflections with *I* > 2σ(*I*)
Graphite monochromator	*R*_int_ = 0.034
Detector resolution: 16.1952 pixels mm^-1^	θ_max_ = 73.5°, θ_min_ = 3.1°
ω scans	*h* = −11→11
Absorption correction: multi-scan (CrysAlis RED; Oxford Diffraction, 2006)	*k* = −15→15
*T*_min_ = 0.766, *T*_max_ = 1.000	*l* = −19→19
24002 measured reflections	

### Refinement

**Table:**

Refinement on *F*^2^	Primary atom site location: structure-invariant direct methods
Least-squares matrix: full	Secondary atom site location: difference Fourier map
*R*[*F*^2^ > 2σ(*F*^2^)] = 0.051	Hydrogen site location: mixed
*wR*(*F*^2^) = 0.136	H atoms treated by a mixture of independent and constrained refinement
*S* = 1.03	*w* = 1/[σ^2^(*F*_o_^2^) + (0.0674*P*)^2^ + 2.3512*P*] where *P* = (*F*_o_^2^ + 2*F*_c_^2^)/3
6572 reflections	(Δ/σ)_max_ < 0.001
461 parameters	Δρ_max_ = 1.04 e Å^−^^3^
9 restraints	Δρ_min_ = −1.22 e Å^−^^3^

### Special details

**Table:**

Geometry. All esds (except the esd in the dihedral angle between two l.s. planes) are estimated using the full covariance matrix. The cell esds are taken into account individually in the estimation of esds in distances, angles and torsion angles; correlations between esds in cell parameters are only used when they are defined by crystal symmetry. An approximate (isotropic) treatment of cell esds is used for estimating esds involving l.s. planes.

### Fractional atomic coordinates and isotropic or equivalent isotropic displacement parameters (Å^2^)

**Table:**

	*x*	*y*	*z*	*U*_iso_*/*U*_eq_	Occ. (<1)
S1	0.25089 (8)	0.29506 (6)	0.04795 (4)	0.02579 (17)	
S2	0.31964 (8)	0.20219 (6)	0.24284 (4)	0.02445 (16)	
O1	0.4742 (2)	0.34505 (16)	0.36255 (13)	0.0236 (4)	
H1O	0.441 (4)	0.342 (3)	0.3195 (17)	0.035*	
O2	0.7068 (2)	0.59223 (17)	0.42660 (14)	0.0248 (4)	
H2O	0.719 (4)	0.538 (3)	0.467 (3)	0.037*	
N1	0.3459 (2)	0.40638 (19)	0.12744 (15)	0.0216 (4)	
H1N	0.334 (4)	0.4674 (17)	0.0787 (14)	0.026*	
N2	0.4002 (2)	0.41587 (19)	0.19707 (15)	0.0203 (4)	
C1	0.3070 (3)	0.3092 (2)	0.13557 (18)	0.0210 (5)	
C2	0.4523 (3)	0.5085 (2)	0.17831 (17)	0.0208 (5)	
H2	0.449270	0.565398	0.119382	0.025*	
C3	0.5149 (3)	0.5276 (2)	0.24467 (17)	0.0191 (5)	
C4	0.5250 (3)	0.4457 (2)	0.33341 (17)	0.0185 (5)	
C5	0.5878 (3)	0.4678 (2)	0.39452 (17)	0.0196 (5)	
H5	0.592987	0.413031	0.454159	0.024*	
C6	0.6428 (3)	0.5688 (2)	0.36936 (18)	0.0200 (5)	
C7	0.6322 (3)	0.6526 (2)	0.28227 (18)	0.0219 (5)	
H7	0.667601	0.722731	0.265441	0.026*	
C8	0.5691 (3)	0.6303 (2)	0.22206 (18)	0.0216 (5)	
H8	0.562034	0.686290	0.163188	0.026*	
C9	0.2662 (3)	0.0836 (2)	0.2270 (2)	0.0285 (6)	
H9A	0.166479	0.106817	0.209163	0.034*	
H9B	0.341362	0.059511	0.176822	0.034*	
C10	0.2594 (3)	−0.0138 (2)	0.31778 (19)	0.0241 (5)	
C11	0.1216 (3)	−0.0316 (2)	0.3794 (2)	0.0279 (6)	
H11	0.031362	0.016945	0.363684	0.034*	
C12	0.1153 (3)	−0.1195 (3)	0.4632 (2)	0.0283 (6)	
H12	0.020831	−0.131504	0.504563	0.034*	
C13	0.2467 (3)	−0.1903 (2)	0.48719 (19)	0.0248 (6)	
H13	0.242045	−0.249847	0.545235	0.030*	
C14	0.3851 (3)	−0.1742 (2)	0.4262 (2)	0.0251 (6)	
H14	0.474899	−0.223164	0.442161	0.030*	
C15	0.3914 (3)	−0.0863 (2)	0.34213 (19)	0.0250 (6)	
H15	0.485897	−0.075124	0.300618	0.030*	
S3	0.73253 (7)	0.34482 (6)	0.03293 (4)	0.02366 (16)	
S4	0.83946 (7)	0.24109 (6)	0.21556 (4)	0.02302 (16)	
O3	0.9879 (2)	0.37367 (16)	0.34167 (13)	0.0256 (4)	
H3O	0.960 (4)	0.372 (3)	0.300 (3)	0.038*	
O4	1.2098 (2)	0.60394 (18)	0.42605 (14)	0.0266 (4)	
H4O	1.225 (4)	0.663 (3)	0.415 (3)	0.040*	
N3	0.8590 (2)	0.44846 (19)	0.10435 (15)	0.0207 (4)	
H3N	0.837 (3)	0.5114 (16)	0.0599 (16)	0.025*	
N4	0.9200 (2)	0.45225 (19)	0.17342 (14)	0.0204 (4)	
C16	0.8112 (3)	0.3530 (2)	0.11379 (17)	0.0196 (5)	
C17	0.9657 (3)	0.5459 (2)	0.16018 (17)	0.0194 (5)	
H17	0.957185	0.607260	0.104159	0.023*	
C18	1.0297 (3)	0.5591 (2)	0.22915 (17)	0.0190 (5)	
C19	1.0386 (3)	0.4737 (2)	0.31632 (18)	0.0194 (5)	
C20	1.0996 (3)	0.4913 (2)	0.38046 (17)	0.0209 (5)	
H20	1.105178	0.433873	0.438900	0.025*	
C21	1.1521 (3)	0.5920 (2)	0.35942 (18)	0.0203 (5)	
C22	1.1458 (3)	0.6781 (2)	0.27344 (18)	0.0215 (5)	
H22	1.182843	0.747006	0.259188	0.026*	
C23	1.0847 (3)	0.6603 (2)	0.21012 (18)	0.0216 (5)	
H23	1.079627	0.718240	0.151889	0.026*	
C24	0.7519 (3)	0.1325 (2)	0.20774 (19)	0.0251 (6)	
H24A	0.646424	0.163586	0.200085	0.030*	
H24B	0.808039	0.107821	0.153245	0.030*	
C25	0.7547 (3)	0.0327 (2)	0.29619 (19)	0.0226 (5)	
C26	0.8227 (3)	−0.0766 (2)	0.2930 (2)	0.0265 (6)	
H26	0.871663	−0.087477	0.234705	0.032*	
C27	0.8194 (3)	−0.1692 (2)	0.3740 (2)	0.0283 (6)	
H27	0.863323	−0.243353	0.370793	0.034*	
C28	0.7522 (3)	−0.1537 (2)	0.4595 (2)	0.0284 (6)	
H28	0.750406	−0.217124	0.515075	0.034*	
C29	0.6876 (3)	−0.0454 (2)	0.4638 (2)	0.0265 (6)	
H29	0.643968	−0.034434	0.522644	0.032*	
C30	0.6862 (3)	0.0475 (2)	0.38258 (19)	0.0234 (5)	
H30	0.638612	0.121017	0.385989	0.028*	
C31	0.7275 (4)	0.9684 (3)	−0.0089 (2)	0.0405 (8)	0.5
H31	0.665510	0.932384	−0.030137	0.049*	0.5
Cl1	0.8790 (2)	1.01634 (17)	−0.10182 (14)	0.0566 (5)	0.5
Cl2	0.6144 (2)	1.09856 (18)	0.01277 (17)	0.0613 (5)	0.5
Cl3	0.7817 (3)	0.87623 (19)	0.08710 (13)	0.0687 (7)	0.5
C31'	0.7275 (4)	0.9684 (3)	−0.0089 (2)	0.0405 (8)	0.5
H31'	0.685078	0.921358	−0.032073	0.049*	0.5
Cl1'	0.6989 (5)	1.0987 (2)	−0.07821 (16)	0.1146 (14)	0.5
Cl2'	0.6300 (2)	0.94980 (17)	0.10809 (11)	0.0516 (4)	0.5
Cl3'	0.9202 (3)	0.8995 (4)	−0.0011 (3)	0.1114 (13)	0.5

### Atomic displacement parameters (Å^2^)

**Table:**

	*U*^11^	*U*^22^	*U*^33^	*U*^12^	*U*^13^	*U*^23^
S1	0.0359 (4)	0.0259 (3)	0.0215 (3)	−0.0055 (3)	−0.0130 (3)	−0.0088 (3)
S2	0.0322 (3)	0.0233 (3)	0.0203 (3)	−0.0048 (3)	−0.0109 (3)	−0.0055 (3)
O1	0.0327 (10)	0.0213 (9)	0.0208 (9)	−0.0084 (8)	−0.0120 (8)	−0.0040 (7)
O2	0.0322 (10)	0.0238 (10)	0.0229 (10)	−0.0075 (8)	−0.0121 (8)	−0.0060 (8)
N1	0.0257 (11)	0.0233 (11)	0.0181 (10)	−0.0035 (9)	−0.0090 (9)	−0.0061 (9)
N2	0.0212 (10)	0.0236 (11)	0.0193 (10)	−0.0020 (9)	−0.0079 (8)	−0.0086 (9)
C1	0.0213 (12)	0.0233 (13)	0.0188 (12)	−0.0019 (10)	−0.0056 (10)	−0.0071 (10)
C2	0.0221 (12)	0.0226 (13)	0.0163 (12)	−0.0017 (10)	−0.0050 (10)	−0.0049 (10)
C3	0.0190 (11)	0.0214 (13)	0.0180 (12)	−0.0023 (10)	−0.0044 (9)	−0.0076 (10)
C4	0.0177 (11)	0.0173 (12)	0.0199 (12)	−0.0010 (9)	−0.0043 (9)	−0.0061 (10)
C5	0.0231 (12)	0.0181 (12)	0.0170 (12)	−0.0007 (10)	−0.0072 (10)	−0.0042 (10)
C6	0.0182 (11)	0.0226 (13)	0.0209 (12)	−0.0017 (10)	−0.0044 (10)	−0.0095 (10)
C7	0.0262 (13)	0.0181 (12)	0.0225 (13)	−0.0062 (10)	−0.0053 (10)	−0.0059 (10)
C8	0.0252 (13)	0.0206 (13)	0.0170 (12)	−0.0047 (10)	−0.0043 (10)	−0.0028 (10)
C9	0.0379 (15)	0.0237 (14)	0.0288 (14)	−0.0070 (12)	−0.0150 (12)	−0.0067 (12)
C10	0.0300 (14)	0.0203 (13)	0.0274 (14)	−0.0056 (11)	−0.0114 (11)	−0.0087 (11)
C11	0.0241 (13)	0.0304 (15)	0.0345 (15)	−0.0015 (11)	−0.0126 (12)	−0.0133 (12)
C12	0.0242 (13)	0.0322 (15)	0.0335 (15)	−0.0089 (11)	−0.0043 (11)	−0.0144 (13)
C13	0.0304 (14)	0.0193 (13)	0.0272 (14)	−0.0072 (11)	−0.0073 (11)	−0.0071 (11)
C14	0.0241 (13)	0.0208 (13)	0.0321 (14)	−0.0013 (10)	−0.0097 (11)	−0.0090 (11)
C15	0.0235 (13)	0.0249 (14)	0.0280 (14)	−0.0068 (11)	−0.0047 (11)	−0.0087 (11)
S3	0.0301 (3)	0.0270 (3)	0.0196 (3)	−0.0083 (3)	−0.0105 (3)	−0.0077 (3)
S4	0.0284 (3)	0.0239 (3)	0.0211 (3)	−0.0101 (3)	−0.0106 (2)	−0.0043 (3)
O3	0.0343 (10)	0.0253 (10)	0.0215 (9)	−0.0117 (8)	−0.0102 (8)	−0.0046 (8)
O4	0.0341 (10)	0.0253 (10)	0.0279 (10)	−0.0056 (8)	−0.0158 (8)	−0.0097 (9)
N3	0.0259 (11)	0.0222 (11)	0.0173 (10)	−0.0073 (9)	−0.0079 (9)	−0.0051 (9)
N4	0.0213 (10)	0.0250 (11)	0.0184 (10)	−0.0049 (9)	−0.0066 (8)	−0.0082 (9)
C16	0.0195 (11)	0.0216 (13)	0.0181 (12)	−0.0032 (10)	−0.0046 (9)	−0.0062 (10)
C17	0.0196 (11)	0.0209 (13)	0.0178 (12)	−0.0032 (10)	−0.0046 (9)	−0.0056 (10)
C18	0.0169 (11)	0.0221 (13)	0.0187 (12)	−0.0025 (9)	−0.0035 (9)	−0.0077 (10)
C19	0.0180 (11)	0.0216 (13)	0.0208 (12)	−0.0030 (10)	−0.0042 (9)	−0.0091 (10)
C20	0.0224 (12)	0.0228 (13)	0.0173 (12)	−0.0020 (10)	−0.0057 (10)	−0.0059 (10)
C21	0.0181 (11)	0.0251 (13)	0.0221 (12)	−0.0002 (10)	−0.0076 (10)	−0.0120 (11)
C22	0.0218 (12)	0.0209 (13)	0.0249 (13)	−0.0047 (10)	−0.0060 (10)	−0.0091 (11)
C23	0.0229 (12)	0.0228 (13)	0.0199 (12)	−0.0034 (10)	−0.0066 (10)	−0.0063 (10)
C24	0.0301 (14)	0.0246 (14)	0.0269 (14)	−0.0097 (11)	−0.0096 (11)	−0.0092 (11)
C25	0.0205 (12)	0.0241 (13)	0.0269 (13)	−0.0082 (10)	−0.0077 (10)	−0.0074 (11)
C26	0.0235 (13)	0.0296 (15)	0.0322 (15)	−0.0084 (11)	−0.0043 (11)	−0.0150 (12)
C27	0.0221 (13)	0.0208 (14)	0.0428 (17)	−0.0037 (10)	−0.0053 (12)	−0.0116 (12)
C28	0.0239 (13)	0.0233 (14)	0.0329 (15)	−0.0068 (11)	−0.0060 (11)	−0.0009 (12)
C29	0.0240 (13)	0.0296 (15)	0.0265 (14)	−0.0068 (11)	−0.0038 (11)	−0.0088 (12)
C30	0.0217 (12)	0.0218 (13)	0.0301 (14)	−0.0048 (10)	−0.0074 (11)	−0.0099 (11)
C31	0.0448 (19)	0.051 (2)	0.0349 (17)	−0.0165 (16)	−0.0082 (14)	−0.0181 (15)
Cl1	0.0650 (12)	0.0526 (11)	0.0532 (11)	−0.0339 (9)	0.0188 (9)	−0.0247 (9)
Cl2	0.0574 (11)	0.0547 (11)	0.0867 (15)	−0.0157 (9)	0.0009 (10)	−0.0467 (11)
Cl3	0.1172 (19)	0.0625 (12)	0.0413 (10)	−0.0488 (13)	−0.0460 (11)	0.0100 (9)
C31'	0.0448 (19)	0.051 (2)	0.0349 (17)	−0.0165 (16)	−0.0082 (14)	−0.0181 (15)
Cl1'	0.262 (5)	0.0535 (14)	0.0460 (12)	−0.084 (2)	−0.0433 (19)	0.0102 (10)
Cl2'	0.0670 (11)	0.0606 (11)	0.0290 (8)	−0.0103 (9)	−0.0098 (8)	−0.0161 (8)
Cl3'	0.0426 (12)	0.209 (4)	0.132 (3)	−0.0222 (17)	−0.0122 (14)	−0.115 (3)

### Geometric parameters (Å, º)

**Table:**

S1—C1	1.680 (3)	O3—H3O	0.78 (4)
S2—C1	1.755 (3)	O4—C21	1.369 (3)
S2—C9	1.816 (3)	O4—H4O	0.75 (4)
O1—C4	1.352 (3)	N3—C16	1.340 (3)
O1—H1O	0.835 (10)	N3—N4	1.376 (3)
O2—C6	1.352 (3)	N3—H3N	0.875 (10)
O2—H2O	0.77 (4)	N4—C17	1.291 (3)
N1—C1	1.327 (3)	C17—C18	1.446 (3)
N1—N2	1.377 (3)	C17—H17	0.9500
N1—H1N	0.881 (10)	C18—C23	1.405 (4)
N2—C2	1.289 (3)	C18—C19	1.417 (4)
C2—C3	1.441 (3)	C19—C20	1.389 (3)
C2—H2	0.9500	C20—C21	1.380 (4)
C3—C8	1.406 (4)	C20—H20	0.9500
C3—C4	1.419 (3)	C21—C22	1.404 (4)
C4—C5	1.388 (3)	C22—C23	1.380 (3)
C5—C6	1.385 (4)	C22—H22	0.9500
C5—H5	0.9500	C23—H23	0.9500
C6—C7	1.410 (4)	C24—C25	1.508 (4)
C7—C8	1.379 (4)	C24—H24A	0.9900
C7—H7	0.9500	C24—H24B	0.9900
C8—H8	0.9500	C25—C30	1.395 (4)
C9—C10	1.512 (4)	C25—C26	1.399 (4)
C9—H9A	0.9900	C26—C27	1.387 (4)
C9—H9B	0.9900	C26—H26	0.9500
C10—C11	1.394 (4)	C27—C28	1.384 (4)
C10—C15	1.403 (4)	C27—H27	0.9500
C11—C12	1.385 (4)	C28—C29	1.386 (4)
C11—H11	0.9500	C28—H28	0.9500
C12—C13	1.388 (4)	C29—C30	1.393 (4)
C12—H12	0.9500	C29—H29	0.9500
C13—C14	1.392 (4)	C30—H30	0.9500
C13—H13	0.9500	C31—Cl3	1.660 (4)
C14—C15	1.386 (4)	C31—Cl1	1.774 (4)
C14—H14	0.9500	C31—Cl2	1.832 (4)
C15—H15	0.9500	C31—H31	1.0000
S3—C16	1.675 (2)	C31'—Cl1'	1.628 (4)
S4—C16	1.749 (3)	C31'—Cl2'	1.773 (4)
S4—C24	1.823 (3)	C31'—Cl3'	1.815 (4)
O3—C19	1.351 (3)	C31'—H31'	1.0000
			
C1—S2—C9	102.06 (13)	C17—N4—N3	116.9 (2)
C4—O1—H1O	108 (2)	N3—C16—S3	121.44 (19)
C6—O2—H2O	108 (3)	N3—C16—S4	114.31 (18)
C1—N1—N2	120.7 (2)	S3—C16—S4	124.25 (16)
C1—N1—H1N	121 (2)	N4—C17—C18	121.1 (2)
N2—N1—H1N	118 (2)	N4—C17—H17	119.4
C2—N2—N1	116.2 (2)	C18—C17—H17	119.4
N1—C1—S1	120.7 (2)	C23—C18—C19	118.1 (2)
N1—C1—S2	114.43 (19)	C23—C18—C17	119.3 (2)
S1—C1—S2	124.88 (16)	C19—C18—C17	122.6 (2)
N2—C2—C3	121.5 (2)	O3—C19—C20	117.1 (2)
N2—C2—H2	119.3	O3—C19—C18	122.7 (2)
C3—C2—H2	119.3	C20—C19—C18	120.2 (2)
C8—C3—C4	117.9 (2)	C21—C20—C19	120.1 (2)
C8—C3—C2	119.8 (2)	C21—C20—H20	120.0
C4—C3—C2	122.3 (2)	C19—C20—H20	120.0
O1—C4—C5	117.3 (2)	O4—C21—C20	117.0 (2)
O1—C4—C3	122.6 (2)	O4—C21—C22	121.8 (2)
C5—C4—C3	120.1 (2)	C20—C21—C22	121.2 (2)
C6—C5—C4	120.6 (2)	C23—C22—C21	118.5 (2)
C6—C5—H5	119.7	C23—C22—H22	120.8
C4—C5—H5	119.7	C21—C22—H22	120.8
O2—C6—C5	122.1 (2)	C22—C23—C18	122.0 (2)
O2—C6—C7	117.3 (2)	C22—C23—H23	119.0
C5—C6—C7	120.6 (2)	C18—C23—H23	119.0
C8—C7—C6	118.5 (2)	C25—C24—S4	108.18 (17)
C8—C7—H7	120.8	C25—C24—H24A	110.1
C6—C7—H7	120.8	S4—C24—H24A	110.1
C7—C8—C3	122.4 (2)	C25—C24—H24B	110.1
C7—C8—H8	118.8	S4—C24—H24B	110.1
C3—C8—H8	118.8	H24A—C24—H24B	108.4
C10—C9—S2	108.38 (18)	C30—C25—C26	118.8 (3)
C10—C9—H9A	110.0	C30—C25—C24	120.5 (2)
S2—C9—H9A	110.0	C26—C25—C24	120.7 (2)
C10—C9—H9B	110.0	C27—C26—C25	120.7 (3)
S2—C9—H9B	110.0	C27—C26—H26	119.7
H9A—C9—H9B	108.4	C25—C26—H26	119.7
C11—C10—C15	119.0 (3)	C28—C27—C26	120.1 (3)
C11—C10—C9	120.3 (2)	C28—C27—H27	119.9
C15—C10—C9	120.7 (3)	C26—C27—H27	119.9
C12—C11—C10	120.4 (3)	C27—C28—C29	119.7 (3)
C12—C11—H11	119.8	C27—C28—H28	120.1
C10—C11—H11	119.8	C29—C28—H28	120.1
C11—C12—C13	120.3 (3)	C28—C29—C30	120.5 (3)
C11—C12—H12	119.9	C28—C29—H29	119.8
C13—C12—H12	119.9	C30—C29—H29	119.8
C12—C13—C14	120.1 (3)	C29—C30—C25	120.1 (2)
C12—C13—H13	120.0	C29—C30—H30	120.0
C14—C13—H13	120.0	C25—C30—H30	120.0
C15—C14—C13	119.7 (2)	Cl3—C31—Cl1	114.0 (2)
C15—C14—H14	120.1	Cl3—C31—Cl2	111.0 (2)
C13—C14—H14	120.1	Cl1—C31—Cl2	104.6 (2)
C14—C15—C10	120.6 (3)	Cl3—C31—H31	109.0
C14—C15—H15	119.7	Cl1—C31—H31	109.0
C10—C15—H15	119.7	Cl2—C31—H31	109.0
C16—S4—C24	101.78 (12)	Cl1'—C31'—Cl2'	113.8 (2)
C19—O3—H3O	108 (3)	Cl1'—C31'—Cl3'	118.7 (3)
C21—O4—H4O	113 (3)	Cl2'—C31'—Cl3'	105.1 (2)
C16—N3—N4	119.5 (2)	Cl1'—C31'—H31'	106.1
C16—N3—H3N	120 (2)	Cl2'—C31'—H31'	106.1
N4—N3—H3N	120 (2)	Cl3'—C31'—H31'	106.1
			
C1—N1—N2—C2	171.8 (2)	C16—N3—N4—C17	179.3 (2)
N2—N1—C1—S1	−176.24 (18)	N4—N3—C16—S3	178.63 (18)
N2—N1—C1—S2	3.8 (3)	N4—N3—C16—S4	−1.7 (3)
C9—S2—C1—N1	−177.9 (2)	C24—S4—C16—N3	176.56 (19)
C9—S2—C1—S1	2.2 (2)	C24—S4—C16—S3	−3.8 (2)
N1—N2—C2—C3	−178.7 (2)	N3—N4—C17—C18	179.1 (2)
N2—C2—C3—C8	−179.3 (2)	N4—C17—C18—C23	177.0 (2)
N2—C2—C3—C4	0.9 (4)	N4—C17—C18—C19	−3.4 (4)
C8—C3—C4—O1	179.0 (2)	C23—C18—C19—O3	179.5 (2)
C2—C3—C4—O1	−1.3 (4)	C17—C18—C19—O3	−0.1 (4)
C8—C3—C4—C5	−0.4 (4)	C23—C18—C19—C20	0.3 (4)
C2—C3—C4—C5	179.4 (2)	C17—C18—C19—C20	−179.3 (2)
O1—C4—C5—C6	179.7 (2)	O3—C19—C20—C21	−179.4 (2)
C3—C4—C5—C6	−0.9 (4)	C18—C19—C20—C21	−0.1 (4)
C4—C5—C6—O2	−178.9 (2)	C19—C20—C21—O4	179.6 (2)
C4—C5—C6—C7	1.9 (4)	C19—C20—C21—C22	−0.3 (4)
O2—C6—C7—C8	179.2 (2)	O4—C21—C22—C23	−179.4 (2)
C5—C6—C7—C8	−1.5 (4)	C20—C21—C22—C23	0.5 (4)
C6—C7—C8—C3	0.2 (4)	C21—C22—C23—C18	−0.3 (4)
C4—C3—C8—C7	0.7 (4)	C19—C18—C23—C22	−0.1 (4)
C2—C3—C8—C7	−179.1 (2)	C17—C18—C23—C22	179.5 (2)
C1—S2—C9—C10	−175.43 (19)	C16—S4—C24—C25	−174.94 (18)
S2—C9—C10—C11	97.7 (3)	S4—C24—C25—C30	57.9 (3)
S2—C9—C10—C15	−81.2 (3)	S4—C24—C25—C26	−123.6 (2)
C15—C10—C11—C12	0.0 (4)	C30—C25—C26—C27	1.4 (4)
C9—C10—C11—C12	−178.9 (2)	C24—C25—C26—C27	−177.1 (2)
C10—C11—C12—C13	0.6 (4)	C25—C26—C27—C28	−1.9 (4)
C11—C12—C13—C14	−1.0 (4)	C26—C27—C28—C29	0.3 (4)
C12—C13—C14—C15	0.8 (4)	C27—C28—C29—C30	1.7 (4)
C13—C14—C15—C10	−0.2 (4)	C28—C29—C30—C25	−2.2 (4)
C11—C10—C15—C14	−0.2 (4)	C26—C25—C30—C29	0.7 (4)
C9—C10—C15—C14	178.7 (2)	C24—C25—C30—C29	179.1 (2)

### Hydrogen-bond geometry (Å, º)

*Cg*1 and *Cg*2 are the centroids of the (C10–C15) and (C25–C30) rings, respectively.

**Table:**

*D*—H···*A*	*D*—H	H···*A*	*D*···*A*	*D*—H···*A*
O1—H1*O*···N2	0.83 (3)	1.91 (3)	2.653 (3)	148 (3)
O3—H3*O*···N4	0.78 (4)	1.97 (4)	2.663 (3)	148 (4)
N1—H1*N*···S3^i^	0.88 (2)	2.46 (2)	3.323 (2)	168 (2)
N3—H3*N*···S1^i^	0.88 (2)	2.53 (2)	3.394 (2)	171 (2)
O2—H2*O*···O4^ii^	0.76 (4)	2.09 (4)	2.841 (3)	170 (4)
O4—H4*O*···*Cg*1^iii^	0.75 (4)	3.00 (4)	3.735 (3)	170 (4)
C27—H27···O2^iv^	0.95	2.59	3.206 (4)	122
C11—H11···*Cg*2^v^	0.95	2.91	3.541 (3)	125
C29—H29···*Cg*1^vi^	0.95	2.87	3.506 (3)	125
C26—H26···Cl1^vii^	0.95	2.75	3.488 (4)	135
C31—H31···Cl2^viii^	1.00	2.66	3.512 (4)	143
C31′—H31′···S1^i^	1.00	2.77	3.579 (4)	139

Symmetry codes: (i) −*x*+1, −*y*+1, −*z*; (ii) −*x*+2, −*y*+1, −*z*+1; (iii) *x*+1, *y*+1, *z*; (iv) *x*, *y*−1, *z*; (v) *x*−1, *y*, *z*; (vi) −*x*+1, −*y*, −*z*+1; (vii) −*x*+2, −*y*+1, −*z*; (viii) −*x*+1, −*y*+2, −*z*.
